# Performance Evaluation of a Commercial Real-Time PCR Method for the Detection of Lupin Traces in Food

**DOI:** 10.3390/foods13040609

**Published:** 2024-02-17

**Authors:** Clara Tramuta, Lucia Decastelli, Francesco Ingravalle, Elisa Barcucci, Sandra Fragassi, Daniela Manila Bianchi

**Affiliations:** 1 Experimental Zooprophylactic Institute of Piedmont, Liguria and Valle d’Aosta, Italian National Reference Center for the Detection of Food Allergens and Substances Causing Food Intolerance (CReNaRiA), 10154 Turin, Italy; lucia.decastelli@izsto.it (L.D.); elisa.barcucci@izsto.it (E.B.); sandra.fragassi@izsto.it (S.F.); manila.bianchi@izsto.it (D.M.B.); 2 Experimental Zooprophylactic Institute of Piedmont, Liguria and Valle d’Aosta, Department Biostatistics, Epidemiology and Risk Analysis (BEAR), 10154 Turin, Italy; francesco.ingravalle@izsto.it

**Keywords:** detection, food matrices, lupin traces, real-time PCR

## Abstract

In accordance with U.S. FDA Foods Program Regulatory Science Steering Committee guidelines, with this study, we optimized and validated a commercial real-time PCR method for the detection of low amounts of lupin in four classes of food matrices: chocolate cookies, ragù, Olivier salad, and barley and rice flour. DNA extracted from blank (true negative) samples artificially contaminated with lupin (*Lupinus albus*) flour at 1000 ppm underwent dilutions with the DNA extracted from the true negative samples up to 0.5 ppm. The limit of detection for real-time PCR was 0.5 ppm in the complex matrices (range, Ct 26–34), making this a specific, robust, and rapid method for lupin allergen detection and labeling. Our validation data support the suitability of this commercially available real-time PCR method for this purpose.

## 1. Introduction

Lupin is increasingly used in food products; its seeds have long been used for human consumption. Lupin consumption has gained popularity with greater awareness of its nutritional value: it is high in protein (30–40%) and dietary fiber (30%) and low in fat (4–7%) [1], alongside the purported health benefits of a lower risk of obesity, diabetes, and heart disease [2,3]. Furthermore, lupin flour is added as an ingredient in products for persons with celiac disease [4] and as a valuable protein source for vegetarians and individuals with milk allergy [5]. In brief, it is commonly found in many food products, such as bakery products, pasta, meat-based products, sauces, and beverages.

Lupin belongs to the family *Fabaceae*, which includes legumes such as peanut and soybean; it can be the source of allergic reactions in sensitive individuals. European legislation demands the declaration of lupin on food labels [6], whereas in the United States, lupin flour and lupin protein are commonly added to gluten-free foodstuff but are not listed as a food allergen, so consumers may not be aware of the potential allergenic risk [7,8].

Lupin food allergy is understudied; data on its prevalence are scant. Perhaps fewer than 4% of consumers who have eaten foods containing lupin have reported an immediate allergic reaction [9,10,11]. However, possible cross-reactions have been identified between lupins and peanuts: 82% of the peanut-sensitized patients in one study were found to also be sensitized to lupin [12]. Allergy to other *Fabaceae* legumes can trigger an allergic reaction after the inadvertent consumption of lupin [13]. A French study involving 5366 patients reported that a percentage ranging from 14.5% to 17% of adults and children, respectively, with peanut allergy are affected by a secondary allergy to lupin [14]. Furthermore, it was observed in the UK that 4% of children and teenagers are allergic to lupin, whereas this percentage was higher (34%) among peanut-allergic children and teenagers. [15]. The main symptoms of lupin allergy are rhinitis and asthma, urticaria and atopic dermatitis, and digestive disorders [16].

It is difficult to detect food allergens like lupin in complex matrices because they are often present only in traces or hidden by the food matrix. Therefore, the development of specific and sensitive methods to detect traces of lupin is essential to ensure consumer health and the quality of life of allergic consumers. DNA-based assays, such as real-time polymerase chain reaction (PCR), have been widely used as an accurate and reliable method for lupin detection in processed foods [17,18], where they are often applied as a complement to enzyme-linked immunosorbent assay (ELISA). Other DNA-based techniques, such as high-resolution melting (HRM) analysis coupled with DNA barcodes and ligation-dependent probe amplification (LPA), have also been used in allergen analysis to identify bean species, including *Lupinus* spp. [18]. An alternative molecular method to traditional protein-based methods is DNA-based allergen-multiplex ligation-dependent probe amplification (MLPA), developed to detect eight allergens (lupin, sesame, soy, hazelnut, peanut, gluten, mustard, and celery) [19]. Protein-based methods, such as high-performance liquid chromatography coupled with tandem MS detection (HPLC–MS/MS) technology, have been proposed for lupin detection in a variety of food products [18]. In addition, sodium dodecyl sulfate–polyacrylamide gel electrophoresis (SDS-PAGE) or native-PAGE have been used to study the immunoreactivity of lupin and soybean allergens in foods affected by thermal processing [20]. To date, three low-molecular proteins (Lup a alpha-, Lup a gamma-, and Lup a delta-conglutin) and two proteins from the PR-10 family have been detected in *Lupinus alba* [21]. Moreover, three allergens have been identified in *Lupinus angustifolius* as being responsible for lupin allergy and are available for diagnostic purposes: the major allergen (Lup an 1), an β-conglutin, which shares sequence similarities with Ara h1, the major peanut (*Arachis hypogaea*) allergen [13], Lup an 3, an LTP, and Lup an 5 from the profilin family.

The study’s aim was to describe the validation of a commercial real-time PCR method according to the U.S. Food and Drug Administration (FDA) guidelines for the validation of analytical methods for nucleic acid sequence-based analysis [22]. Specifically, we report the validation of a sensitive, specific, and robust real-time PCR method commercially available for the detection of *Lupinus albus* traces in food products. The main utility of the method resides in its application by official food safety control laboratories in the interest of compliance with regulations and to protect food-allergic consumers.

## 2. Materials and Methods

### 2.1. Food Samples

Food matrices for method validation were selected from four categories: ready-to-eat, meat preparation, bakery products, and grains or milling products. The method was tested on four matrices: chocolate cookies, ragù meat sauce, ready-to-eat Olivier salad, rice and barley flour. To guarantee that the true negative blank samples were “lupin free”, the ingredients for their preparation came from primary production as reported in a previous study [23]. Briefly, the chocolate cookies were prepared with corn field harvest flour, commercial cocoa beans, farm-fresh eggs, and commercial oil and sugar. Rice and barley in grains were used to prepare the rice and barley flour. The Olivier salad was prepared with farm-fresh eggs, commercial oil, and carrots and peas from a private house garden. Finally, ragù was made with beef muscle taken at slaughtering, while tomato, onion, and carrots were collected from a private house garden.

A portion of the true negative blank samples was contaminated with lupin (*Lupinus albus*) flour at 1000 mg/kg (ppm).

### 2.2. DNA Extraction

DNA extraction was performed on 10 test portions (replicates) from true negative blank samples and all spiked samples according to the commercial ION Force FAST (Generon, San Prospero, MO, Italy) kit. All protocol procedures were performed according to previously described protocols [23] and the manufacturer’s instructions.

### 2.3. Real-Time PCR

A total volume of 20 µL of PCR Master mix (5 µL of DNA extract + 15 µL of reaction mix) was amplified using the commercial kit RT-PCR SPECIALfinder MC Lupin (Generon) on a CFX96 real-time PCR system (Bio-Rad, Richmond, CA, USA), following the previously described conditions [23].

### 2.4. Specificity, Sensitivity, Robustness, and Repeatability

The specificity of the primers and probes was compared in silico by confronting the sequences in the NCBI BLASTn database of non-target matrices. We used (1) non-target allergens: almond (*Prunus dulcis*), barley (*Hordeum vulgare*), Brazil nut (*Bertholletia excelsa*), buckwheat (*Fagopyrum esculentum*), cashew (*Anacardium occidentale*), celeriac (*Apium graveolens*), celery (*Apium graveolens*), coconut (*Cocos nucifera*), durum wheat (*Triticum durum*), einkorn (*Triticum monococcum*), hazelnut (*Corylus avellana*), Turanicum wheat (*Triticum turanicum)*, macadamia nut (*Macadamia integrifolia*), mustard (*Sinapis*), oats (*Avena sativa*), peanut (*Arachis hypogaea*), pecan nut (*Carya illinoinensis*), pine nut (*Pinus pinea*), pistachio (*Pistacia vera*), rye (*Secale cereale*), sesame (*Sesamum indicum*), soft wheat (*Triticum aestivum*), soy (*Glycine max*), walnut (*Juglans*), clam (*Venus gallina*), hake (*Merluccius merluccius*), lobster (*Nephrops norvegicus*), mussel (*Mytilus edulis*); prawn (*Penaeus vannamei*), salmon (*Oncorhynchus kisutch*), sea bream (*Sparus aurata*), squid (*Loligo edulis*), and yellowfin tuna (*Thunnus albacares*); (2) animal species: bison (*Bison*), wild boar (*Sus scrofa*), buffalo (*Bubalus bubalis*), cow (*Bos taurus*), chicken (*Gallus domesticus*), donkey (*Equus asinus*), duck (*Anas platyrhyncos*), goat (*Capra aegagrus hircus*), goose (*Anser anser domesticus*), horse (*Equus caballus*), quail (*Coturnix coturnix*), rabbit (*Oryctolagus cuniculus*), sheep (*Ovis aries*), swine (*Sus scrofa domesticus*), and turkey (*Meleagris*); and (3) vegetable species: apple (*Malus*), apricot (*Prunus armeniaca*), arugula (*Eruca sativa*), eggplant (*Solanum melongena*), banana (*Musa*), basil (*Ocimum basilicum*), bean (*Phaseolus vulgaris*), black pepper (*Piper nigrum*), blackberry (*Rubus* subg. *Rubus*), broccoli (*Brassica oleracea* var. *italica*), brussels sprouts (*Brassica oleracea* var. *gemmifera*), black cabbage (*Brassica oleracea* L. var. *acephala sabellica*), cacao (*Theobroma cacao*), carrot (*Daucus carota*), cauliflower (*Brassica oleracea* var. *botrytis*), chard (*Beta vulgaris*), cherry (*Prunus avium*), chestnut (*Castanea sativa*), chickpea (*Cicer arietinum*), clementine (*Citrus clementina*), corn (*Zea mays*), cucumber (*Cucumis sativus*), currant (*Ribes*), fennel (*Foeniculum vulgare*), fig (*Ficus carica*), flax (*Linum usitatissimum*), garlic (*Allium sativum*), ginger (*Zingiber officinale*), grapefruit (*Citrus paradisi*), grapevine (*Vitis vinifera*), kiwi (*Actinidia deliciosa*), laurel (*Laurus nobilis*), lemon (*Citrus limon*), lentil (*Lens culinaris*), mahaleb (*Prunus mahaleb*), marrow (*Cucurbita*), mushroom (*Agaricus bisporus*), mango (*Mangifera indica*), marjoram (*Origanum majorana*), olive (*Olea europaea*), onion (*Allium cepa*), orange (*Citrus sinensis*), oregano (*Origanum vulgare*), parsley (*Petroselinum crispum*), pea (*Pisum sativum*), peach (*Prunus persica*), pear (*Pyrus*), pepper (*Capsicum annuum*), pineapple (*Ananas comosus*), pink peppercorn (*Schinus terebinthifolius*), plum (*Prunus domestica*), pomelo (*Citrus maxima*), poplar (*Populus*), poppy (*Papaver*), potato (*Solanum tuberosum*), radish (*Raphanus sativus*), rapeseed (*Brassica napus*), raspberry (*Rubus idaeus*), red cabbage (*Brassica oleracea* var. *capitata*), rice (*Oryza sativa*), saffron (*Crocus sativus*), savoy cabbage (*Brassica oleracea* var. *sabauda*), shallot (*Allium ascalonicum*), spinach (*Spinacia oleracea*), strawberry (*Fragaria*), sunflower (*Helianthus annuus*), tangerine (*Citrus reticulata*), tarragon (*Artemisia dracunculus*), thyme (*Thymus vulgaris*), tomato (*Solanum lycopersicum*), and turnip greens (*Brassica rapa*). The specificity of the PCR primers was tested by amplifying the DNA extracted from the true negative blank samples processed in 40 replicates (10 for each of the 4 food matrices) containing principally corn (*Zea mays*), egg (*Gallus domesticus*), cocoa (*Theobroma cacao*), rice (*Oryza sativa*), barley (*Hordeum vulgare*), carrot (*Daucus carota*), peas (*Pisum*), beef (*Bos taurus*), and tomato (*Solanum lycopersicum*). The extracted DNA of the following non-target matrices was also tested experimentally in celery (*Apium graveolens*), almonds (*Prunus dulscis*), soy (*Glycine max*), mustard (*Sinapis alba*), and onion (*Allium cepa*) to determine method specificity.

To estimate method sensitivity, DNA extracted from the spiked samples (10 replicates of each matrix for a total of 40 replicates at 1000 ppm) was diluted to a concentration of 100, 10, 5, and 0.5 ppm, adding an opportune volume of true negative blank sample-extracted DNA, and then, they were tested with the commercial kit Real-Time PCR SPECIALfinder MC Lupin (Generon). The sensitivity of the real-time PCR was determined by testing the correspondence between 10-fold serial dilutions of DNA extracted from spiked chocolate cookie samples diluted to a theoretical concentration of 500, 50, 5, and 0.5 ppm. All PCR reactions were conducted using a positive control and a no template control (NTC) run in parallel.

ANOVA was performed to determine whether there were differences between the matrices in the Ct values detected at the LOD and 10 × LOD concentrations. The factors considered in this model were the concentration, the matrix, and the interaction between these two variables. The Shapiro–Francia test was used to test the normality of the Ct values.

To determine method robustness, 20 µL of PCR master mix containing 5 µL DNA extracts of the spiked chocolate cookies was amplified on a StepOnePlus Instrument (Applied Biosystems, Foster City, CA, USA). For this purpose, we applied the *t*-test for paired results.

The relative standard deviation repeatability (RSDr) was statistically calculated on 40 replicates at the limit of detection (LOD) concentration, using the same samples and the same laboratory instruments and performed by the same technical staff.

## 3. Results

### Specificity, Sensitivity, Robustness, and Repeatability

The specificity of the primers and probes was verified by checking the homology of the DNA sequences of the allergens, and animal and vegetable species in the NCBI BLAST database. From the in silico test came out that non-target matrices were not affected by cross-reaction. Evaluation of method specificity revealed high performance of the test: no aspecific signals were present in the 40 PCR tests performed on true negative blank samples and in the non-target matrices celery (*Apium graveolens*), almonds (*Prunus dulscis*), soy (*Glycine max*), mustard (*Sinapis alba*), and onion (*Allium cepa*).

**Figure 1:**
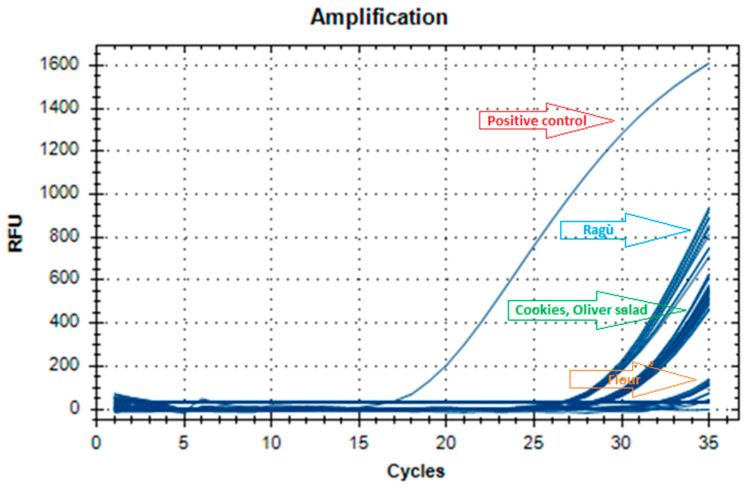
Amplification curves of the real-time PCR analysis of DNA extracts from the spiked samples to the LOD concentration corresponding to 0.5 ppm (total of 40 replicates, positive control, and no template control).

**Figure 2:**
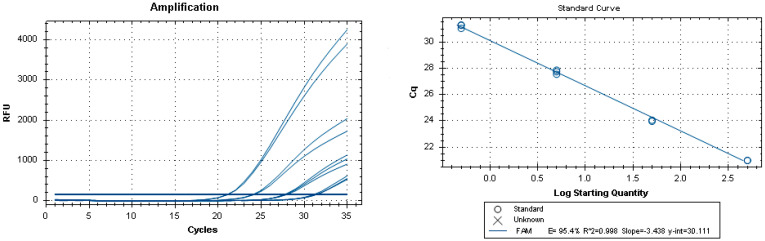
Standard curves obtained using DNA serially diluted 10-fold (500, 50, 5, and 0.5 ppm) from the spiked cookie samples.

To investigate method sensitivity, 100, 10, 5, and 0.5 ppm dilutions of the DNA extracts from the spiked samples were analyzed. The LOD was 0.5 ppm in all food matrices, thus confirming 100% method sensitivity (Table 1, Figure 1). During the analytical sessions, it was found that a range from 26 to 34 Ct (cycle threshold) was significant for samples at the LOD concentration (Figure 2, Table 1). Table 2 presents the real-time PCR Ct of the DNA extracted from the spiked samples diluted to a concentration of 5 ppm with the true negative blank sample-extracted DNA, corresponding to 10 × LOD. Standard curves generated using DNA samples serially diluted ten-fold from spiked chocolate cookie samples (500, 500, 50, 50, 5, 5, 5, 0.5, 0.5, and 0.5 ppm) as the template in the commercial real-time PCR are shown in Figure 2 (Ct range: 21–31.30). All amplifications showed a positive signal in the PCR positive control (Table 1 and Table 2, Figure 1) and a negative signal in the NTCs, indicating the absence of contamination with reagents or primer–dimer formation (no Ct value given by the instruments) (Figure 1).

The statistical distribution of the Ct values for each combination of matrix and concentration fitted the normal distribution satisfactorily: all of the *p*-values were greater than 0.05.

Parametric ANOVA fitted to the Ct values showed statistical significance for the concentration (*p* < 0.001) and the matrix (*p* < 0.001) and their interaction (*p* < 0.001). Table 3 presents a pairwise comparison with the *p*-value (*p*) and significance.

The robustness test revealed no statistically significant differences between the two thermal cyclers (mean Ct, 28.33; range, 28–28.59) (Table 4), demonstrating that the PCR assay is robust.

The results of the performance evaluation study showed a high level of repeatability as RSDr did not exceed 25% for the blank or spiked sample sessions. In contrast, the *t*-test for paired data showed that the difference between Ct obtained with the StepOnePlus Instrument and with the CFX96 real-time PCR system is not negligible: *p*-value = 0.0351.

## 4. Discussion

Method validation represents the activity involved in demonstrating or confirming that an analytical procedure is suitable for use; this implies a series of analytical sessions to confirm the performance of the test. The U.S. FDA Foods Program Regulatory Science Steering Committee (RSSC) has published guidelines [22] describing the technical characteristics that must be checked. Our study showed that the real-time PCR assay for the detection of lupin intended as an undeclared food allergen was fully and positively validated. The present protocol was also successfully accredited according to ISO 17025:2008 [24] requirements.

In 2019, the Italian Ministry of Health designated the Food Safety Laboratory of our institution as the Italian national reference center for the detection of food allergens and substances causing food intolerance (CReNaRiA). It works within the Official Laboratories network, supporting technical competency and sharing newly developed methods with the aim of guaranteeing uniformity of performance for the national competent authority in the field of food allergens.

This real-time PCR protocol was validated and accredited with the aim of implementing the use of specific and sensitive methods for the detection of lupin DNA in foods. The specificity test showed that the method can discriminate between lupin DNA and DNA from closely related species of food ingredients such as meat, eggs, legumes, cereals, and vegetables. The method’s sensitivity is 0.5 ppm for chocolate cookies, ragù meat sauce, ready-to-eat Olivier salad, and rice and barley flour, and it is compatible with current international regulations. Although the chocolate cookie matrix is known to inhibit PCR because of lipids (e.g., cocoa, oil, and eggs), the commercial real-time PCR showed an LOD of 0.5 ppm in the ten replicates. Anyway, very similar mean and median Ct values were observed in this matrix spiked to the LOD and ten-fold the LOD compared to the other food matrices selected. Therefore, the test is suitable for *Lupinus albus* detection in official food samples.

The analytical methods used most often in own-check private laboratories and other official control laboratories are ELISA-based and PCR-based approaches. The commercial real-time PCR assay has the advantage that it is highly specific and sensitive besides being considerably faster (results within less than 50 min) and easier to execute than other protein-based methods. While LC-MS/MS assays are being further refined, they require very expensive equipment and well-trained personnel [25]. Moreover, European legislation on food allergens does not exclude any of the analytical approaches from the exploitable assays to determine hidden allergens in food samples. Allergenic substances are largely proteins. PCR methods based on the detection of specific DNA sequences encoding proteins are appreciated worldwide because of the minor effect that food processing has on DNA compared with the effect of processing on the expressed protein [16,26,27].

Correct management and cleaning of processing environments and equipment are of fundamental importance in the food industry to minimize the risk of cross-contamination with ingredients that cause allergies. Furthermore, correct food labeling is meant to protect the allergic consumer by stating the allergenic ingredients and the voluntary indications such as “may contain” wordings if the risk of cross-contamination is unavoidable [28,29,30].

## 5. Conclusions

Based on our results, the present commercial real-time PCR method proved suitable for lupin detection and demonstrated specificity, sensitivity, and robustness. Methods that are both specific and sensitive for the detection of allergens in foods are needed to protect allergen-sensitive consumers and to ensure compliance with allergen labeling regulations by food manufacturers. Official food safety laboratories apply analytical methods validated prior to use and validation is performed out of necessity in complex matrices.

## Figures and Tables

**Table 1:** Real-time PCR cycle threshold values to the LOD concentration corresponding to 0.5 ppm (10 replicates of each matrix).

Replicates	Cookies (Ct)	Ragù (Ct)	Flour (Ct)	Olivier Salad (Ct)
1	28.23	26.79	32.39	28.89
2	28.58	26.87	33.53	28.51
3	28.97	26.83	32.25	28.56
4	28.87	26.76	32.58	28.38
5	28.94	27.01	32.02	28.78
6	28.30	26.91	32.02	28.14
7	29.04	26.72	32.46	28.57
8	28.95	26.64	32.40	28.70
9	28.78	26.42	34.55	28.51
10	28.48	26.63	33.81	28.34
Mean	28.71	26.76	32.8	28.54
SD	0.29	0.17	0.86	0.22
Median	28.82	26.77	32.43	28.53
Minimum	28.23	26.42	32.02	28.14
Maximum	29.04	27.01	34.55	28.89
Positive control	16.62	16.62	16.62	16.62

**Table 2:** Real-time PCR cycle threshold values to the 10 × LOD concentration corresponding to 5 ppm (10 replicates of each matrix).

Replicates	Cookies (Ct)	Ragù (Ct)	Flour (Ct)	Olivier Salad (Ct)
1	27.62	25.31	30.23	26.14
2	28.06	25.28	30.13	25.75
3	28.13	24.50	29.60	25.61
4	27.38	24.37	30.17	25.87
5	28.61	24.84	29.92	25.37
6	28.15	24.53	29.67	25.45
7	27.52	25.14	29.57	25.79
8	28.18	25.63	30.06	25.79
9	28.21	24.58	29.35	26.03
10	28.03	24.77	29.30	26.18
Mean	27.99	24.89	29.8	25.8
SD	0.37	0.42	0.34	0.27
Median	28.1	24.80	29.79	25.79
Minimum	27.38	24.37	29.30	25.37
Maximum	28.61	25.63	30.23	26.18
Positive control	16.23	16.23	16.23	16.23

**Table 3:** Pairwise comparison with the *p*-value (*p*) and significance with ANOVA (with Bonferroni correction).

Pairwise Comparisons			LOD		10 × LOD	
			*p*-Value (*p*)		*p*-Value (*p*)	
Cookies	vs.	Olivier salad	1.0000		0.0000	**
Cookies	vs.	Flour	0.0000	**	0.0000	**
Cookies	vs.	Ragù	0.0000	**	0.0000	**
Olivier salad	vs.	Flour	0.0000	**	0.0000	**
Olivier salad	vs.	Ragù	0.0000	**	0.0000	**
Flour	vs.	Ragù	0.0000	**	0.0000	**

** *p* < 0.01.

**Table 4:** Cycle threshold values of real-time PCR. To determine the method’s robustness, we analyzed DNA extracts from the spiked cookie samples to the LOD concentration corresponding to 0.5 ppm (10 replicates) in two thermal cyclers.

Replicates	Cookies (Ct) StepOnePlus Instrument	Cookies (Ct) CFX96 Real-Time PCR System	Difference
1	28.57	27.62	0.95
2	28.34	28.06	0.28
3	28.26	28.13	0.13
4	28.43	27.38	1.05
5	28.54	28.61	−0.07
6	28.34	28.15	0.19
7	28.08	27.52	0.56
8	28.12	28.18	−0.06
9	28	28.21	−0.21
10	28.59	28.03	0.56
Mean	28.33	27.99	0.34
SD	0.21	0.37	0.43
Median	28.34	28.1	0.24
Minimum	28	27.38	−0.21
Maximum	28.59	28.61	1.05
Positive control	16.52	16.62	

## Data Availability

Data is contained within the article.
